# Medical Colleges

**Published:** 1877-08-20

**Authors:** 


Medical Colleges.—We give place to the following extract of a private letter on this subject: "Your brief editorial on the question, 'Where to send our Medical Students,' meets my hearty approval. First-class schools alone should be sustained by the profession. I have some views on this subject which, if time permits, I will send you for publication, perhaps by your next issue."

While it is peculiarly the province of medical journalists to advocate the ethics of our profession, and especially to encourage a high standard of medical education, it is equally the duty of every member of the profession to aid in the work. We are gratified, therefore, at the reception of letters like the above, and shall be pleased to give place to the promised communication, if it comes.

